# Synthesis and physical properties of brominated hexacene and hole-transfer properties of thin-film transistors†

**DOI:** 10.1039/c7ra13632c

**Published:** 2018-04-10

**Authors:** Motonori Watanabe, Takaaki Miyazaki, Toshinori Matsushima, Junko Matsuda, Ching-Ting Chein, Masahiko Shibahara, Chihaya Adachi, Shih-Sheng Sun, Tahsin J. Chow, Tatsumi Ishihara

**Affiliations:** International Institute for Carbon Neutral Energy Research, Kyushu University 744 Motooka, Nishi-ku, Fukuoka 819-0395 Japan mwata@i2cner.kyushu-u.ac.jp; Education Center for Global Leaders in Molecular Systems for Devices, Kyushu University 744 Motooka Nishi-ku Fukuoka 819-0395 Japan; Center for Organic Photonics and Electronics Research (OPERA), Kyushu University 744 Motooka, Nishi-ku Fukuoka Japan; Japan Science and Technology Agency (JST), ERATO, Adachi Molecular Exciton Engineering Project 744 Motooka, Nishi-ku Fukuoka 819-0395 Japan; Institute of Chemistry, Academia Sinica No. 128, Academia Road Sec 2, Nankang Taipei 11529 Taiwan; Division of Natural Sciences, Faculty of Science and Technology, Oita University Dannoharu 700 Oita Japan; Department of Applied Chemistry, Faculty of Engineering, Kyushu University Nishi-ku Fukuoka 819-0395 Japan; Department of Chemistry, Tunghai University Taichung 40704 Taiwan

## Abstract

A halide-substituted higher acene, 2-bromohexacene, and its precursor with a carbonyl bridge moiety were synthesized. The precursor was synthesized through 7 steps in a total yield of 2.5%. The structure of precursor and thermally converted 2-bromohexacene were characterized by solid state NMR, IR, and absorption spectra, as well as by DFT computation analysis. It exhibited high stability in the solid state over 3 months, therefore can be utilized in the fabrication of opto-electronic devices. The organic thin-film transistors (OFETs) were fabricated by using 2-bromohexacene and parent hexacene through vaccum deposition method. The best film mobility of 2-bromohexacene was observed at 0.83 cm^2^ V^−1^ s^−1^ with an on/off ratio of 5.0 × 10^4^ and a threshold of −52 V, while the best film mobility of hexacene was observed at 0.076 cm^2^ V^−1^ s^−1^ with an on/off ratio of 2.4 × 10^2^ and a threshold of −21 V. AFM measurement of 2-bromohexacene showed smooth film formation. The averaged mobility of 2-bromohexacene is 8 fold higher than the non-substituted hexacene.

## Abstract 2

A halide-substituted higher acene, 2-bromohexacene, and its precursor with a carbonyl bridge moiety were synthesized.

## Graphical abstract

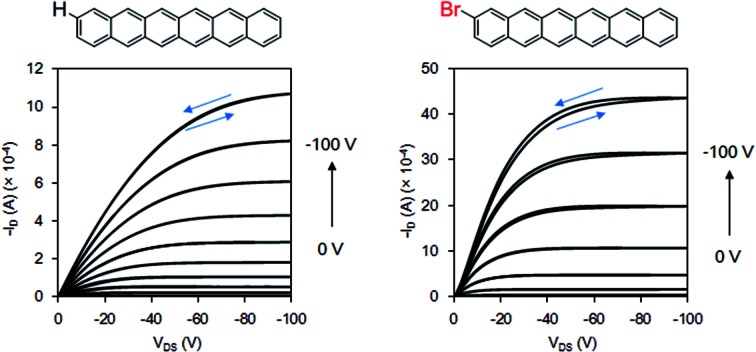

## 1. Introduction

Acenes are amongst the most representative hydrocarbons for analysing the physical properties of polycyclic hydrocarbon materials.^1^ Along with the increase in the number of aromatic benzene rings, acenes exhibit a reduction of both the HOMO–LUMO gap and the reorganisation energy.^2^ The chemistry of acenes higher than pentacene, particularly their open-shell characteristics^3^ and high charge transport properties, has attracted considerable attentions. Hence, these compounds and their analogs^4^ such thieonoacene based semiconductor^5^ are suitable for use in organic electronic devices such as organic field-effect transistors.^6–8^ The extended π-conjugation of higher acenes also induce an interesting phenomenon of singlet fission that can be used on light harvesting.^9^ Bulky substituents can enhance the thermal and photo-stability of acenes by lowering the radical characteristic in the ground state.^10^ The isolation of higher acenes, from hexacene to nonacene and derivatives, has been achieved by applying this strategy. The modification of physical properties of acenes in the solid state requires a crystal engineering approach; however, their isolation steps are either difficult or tedious in order to obtain qualified structures due to their high thermal and light sensitivity in solutions.^11,12^ To overcome the difficulty, stable precursors of acenes are prepared first, which can then be converted to the corresponding acenes quantitatively in demand through either a thermal or a photo-driven process.^13,14^ The synthesis of nonacene derivatives has been achieved by this approach utilising a diketone precursor through photo-induced transformation.^15^ Recently, the dimer structure of heptacene was converted to heptacene *via* a thermal retro-cyclization reaction, therefore showing its feasibility for further processes.^16^ The precursor method can be used to produce higher acenes in large quantity that is required to become usable materials. Acenes have certain valuable potential applications, such as organic semi-conductors,^17^ singlet fission materials,^18,19^ and organic biradical sources.^15^

Previously, our group has developed the method of producing higher acene molecules from either monoketone precursors^20^ or from diethylketomalonate precursors.^21^ Both types of precursor can be cleanly converted to hexacene either thermally or photo-chemically. In addition, our group has generated halide-substituted tetracene^22^ and pentacene^23^ from their corresponding monoketone precursors. They were used successfully as the semiconductors in electronic devices. In these devices, single crystal bromopentacene device exhibits a significant superior hole mobility (>5 cm^2^ V^−1^ s^−1^) to the parent pentacene (1.4 cm^2^ V^−1^ s^−1^). Such a high performance is comparable with other related materials, such as triisopropylsilylethynylpentacene (>1 cm^2^ V^−1^ s^−1^),^24–26^ alkylated dinaphtho[2,3-*b*:2′,3′-*f*]thieno[3,2-*b*]thiophenes (>10 cm^2^ V^−1^ s^−1^),^27,28^ and the crystal of tetracene analogous of rubrene (>18 cm^2^ V^−1^ s^−1^).^29,30^ In previous reports, the charge mobility of hexacene and derivatives were measured in the devices either made with single crystals,^21^ or with crystalline thin films prepared through solution method.^22,31^ However, the physical properties and transistor characteristics of hexacene thin film that is prepared by vacuum deposition method have not yet been reported.

**Fig. 1:**
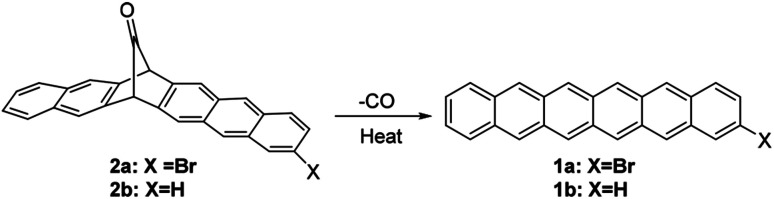
The synthesis of 2-bromohexacene from its monoketone precursor.

It is believed that the bromine substituent can provide a suitable size to improve crystal packing. Judged by the past high-performance of brominated tetracene and pentacene, it is therefore in demand to explore the possibility of brominated analogue of hexacene. In this regard, the brominated analogue of hexacene, *i.e.*, 2-bromohexacene (1a), is synthesized and its charge-transport property is examined. Precursor 2a is cleanly converted to 1a by thermal decomposition, and 1a exhibits high thermal stability in the dark over 90 days (Fig. 1). This is the first example of the charge-transport property of a stable vacuum-deposited thin-film of hexacene and halogenated hexacene for electronic devices.

## 2. Results and discussion

### 2.1. Synthesis

**Scheme 1:**
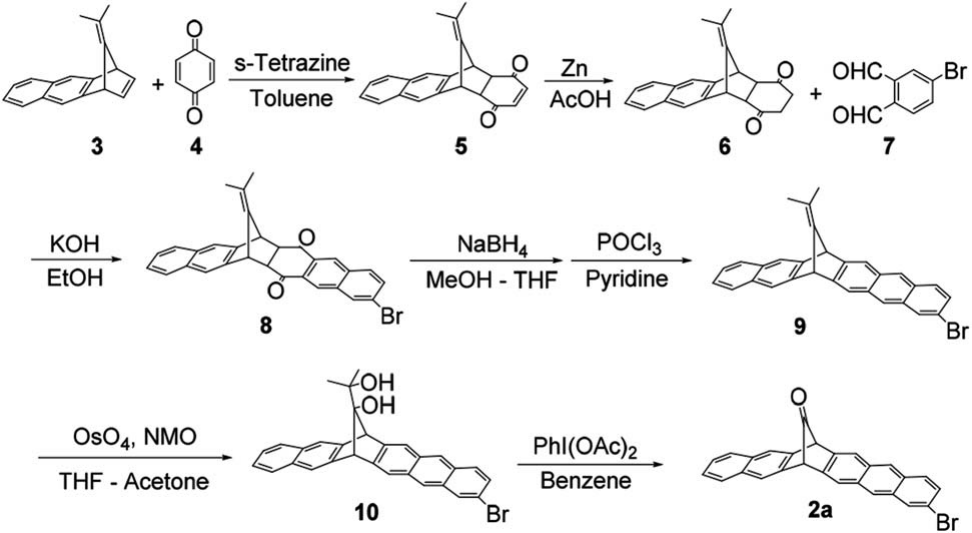
Synthetic route of 2a.

The synthesis of 2a is shown in Scheme 1. Dimethylfulvene derivative 3 and benzoquinone 4 were treated with *s*-tetrazine, affording diketone 5 as an *endo*–*exo* mixture, then the double bond of 5 was reduced using zinc in acetic acid, affording adduct 6. The aldol reaction of 6 with dialdehyde 7 afforded diketone 8 as an *endo* compound in 17% yield (from 3, three steps). Pure *endo*-8 was crystallized probably due to steric influence by the bromo-substituent. It was reduced by NaBH_4_, and further treatment with POCl_3_/pyridine afforded aromatic compound 9 in 48% yield (from 8, two steps). The *exo*-double bond was treated with OsO_4_ to give a diol, followed by treatment with PhI(OAc)_2_ to give desired 2a in 31% yield. The total yield was 2.5% in 7 steps.

The absorption spectrum of 2a in 1,2,4-trichlorobenzene exhibited characteristic ^1^A–^1^L_a_ transitions of the anthracene chromophore at 353, 371, 391 and 408 nm, with vibrionic progressions, which were red-shifted from the peaks of parent 2b at 350, 368 and 391 nm in the 1,2,4-trichlorobenzene solution (Fig. S1†). When the solution of 2a was heated at 230 °C, the solution changed from colorless to pale-green, which exhibited characteristic acene vibration absorption bands at 573, 623 and 679 nm. However, this colour changed to yellow within a few minutes owing to the dimerization or oxidation of 1a.

### 2.2. Physical properties

The absorption maximum in the visible range was observed at 679 nm, whereas parent hexacene 1b exhibited the peak maximum at 675 nm in 1,2,4-trichlorobenzene (Fig. S1†). The peaks were red-shifted from those of parent hexacene 1b by 4 nm, indicating the reduction of HOMO–LUMO gap of 1a by the bromo substituent. This phenomenon has similarly been observed in the pairs of tetracene (473 nm in THF) and 2-bromotetracene (477 nm in THF),^22^ as well as pentacene (575 nm in THF) and 2-bromopentacene (578 nm in THF).^23^ To compare with the reported hexacene analogues, the peak maximum was shown to be red-shifted from that of pentaceno[2,3-*b*]thiophene (640 nm in *o*-DCB)^30^ due to the increase of aromaticity. In comparison with other substituent group effect, however, it showed a blue shift from those of tricyclohexylsilylethynyl-octafluorohexacene (725 nm),^31^ tri-*tert*-butylsilylethyny-hexacene (738 nm),^32,33^ and trialkylsilylethynyl-azahexacene (825–842 nm in hexane)^34^ due to the electron-donating effect of the trialkylsilylethynyl acetylene units.

**Fig. 2:**
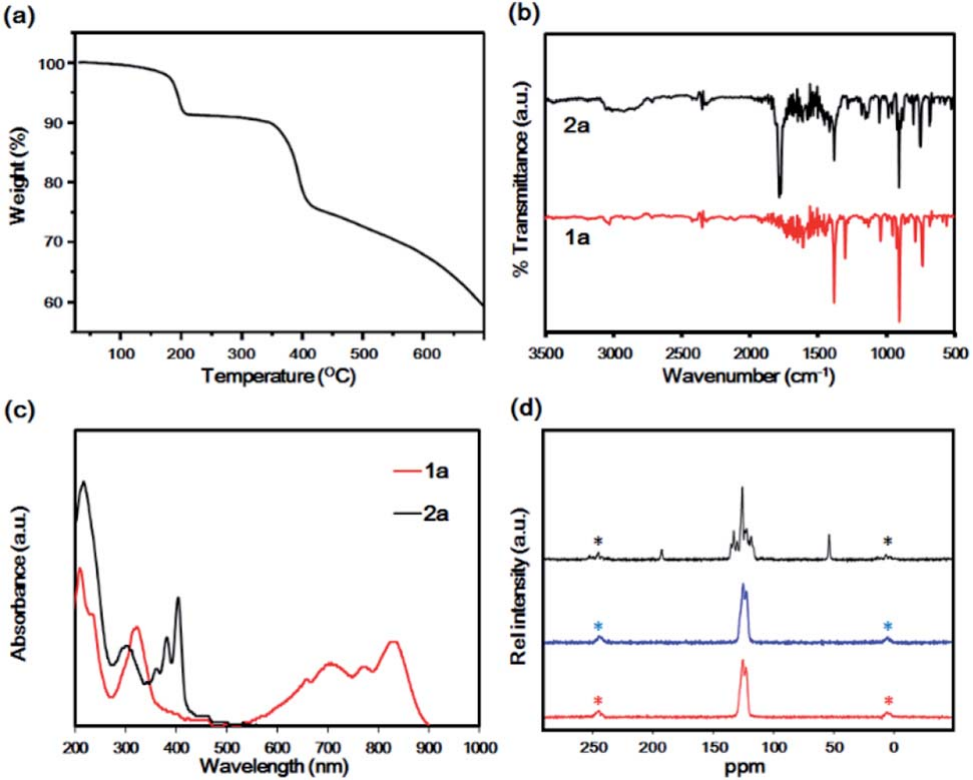
(a) TGA profile of 2a under nitrogen. Heating rate was 10 °C min^−1^. (b) IR spectra of 2a (black) and after heating at 230 °C for conversion to 1a (red) under ambient conditions. (c) Reflectance absorption spectra of drop-casted 2a on a quartz plate (black) and after heating at 230 °C for conversion to 1a (red) under ambient conditions. (d) Solid-state ^13^C CP-MAS spectra of 2a (black), 1a (blue) and 1a after 90 days in the dark (red). The asterisk denotes the spinning side band.

In the thermal gravimetric analysis (TGA) profile of 2a, the first weight loss (8.5%, calcd 6.5%) was observed at approximately 200 °C to generate 1a. The thermal weight loss profile did not change up to 360 °C. Then it was followed by the second weight loss caused by the vaporisation as well as the decomposition of 1a at temperatures greater than 400 °C (Fig. 2a). Decarbonylation at 200 °C was confirmed by infrared (IR) spectroscopy, which revealed the disappearance of the characteristic C=O stretching band at 1786 cm^−1^ after heating at 230 °C (Fig. 2b). The high-resolution FAB-MS spectrum revealed a molecular ion signal at *m*/*z* 406.0387 (M+, calcd 406.03571, error = +7.4 ppm), which corresponded to 1a. The purity was also confirmed by correct elemental analysis. The absorption spectra of 2a, fabricated by drop-casting a saturated chlorobenzene solution on a quartz substrate, exhibited a similar vibration structure to that of 2a in solution, with characteristic peaks at 403, 380 and 364 nm, indicative of the fabrication of film of 2a. In contrast, after heating at 230 °C under nitrogen, a broad band was observed at 500–900 nm, and the peaks at 360–400 nm, which are characteristic of the anthracene moiety, disappeared.

These structures were different from that of 1a in solution. Film 1a exhibited characteristic peaks at 833 and 776 and at 708 and 661 nm. These peaks exhibited the appearance of Davydov splitting effect.^20,21,35^ The same pattern appeared in other related acene structures in the solid state, including parent 1b, at 840 nm, 765 nm, 708 nm and 654 nm.^20^ The first bands at 833 and 775 nm corresponded to the 0–0 band, and those at 708 and 654 nm corresponded to the 0–1 band (Fig. 2c). The high thermal stability of 1b in the solid state was confirmed by solid-state NMR that maintained invariant over 1 month.^13^ Comparing it with heptacene under a similar situation, the latter dimerised slightly after 1 month.^16^ To verify the thermal stability of 1a, ^13^C CP-MAS NMR spectra were recorded for monitoring the variation of the carbon skeleton. Compound 2a exhibited three main peaks at 193.2 (bridge position of C=O), 137–114 (aromatic carbon atoms) and 54.4 ppm (bridgehead tertiary carbon atom). After the conversion to 1a, the spectrum exhibited aromatic carbon peaks at 125.7 and 122.9 ppm only, indicating a quantitative transformation. After maintaining 1a for 90 days in the dark under air atmosphere, no changes were observed in the CP-MAS spectrum, indicative of the high thermal stability of 1a (Fig. 2d). This high thermal stability can be compared with the reported property of hexacene (>1 month in dark)^20^ and tricyclohexylsilylethynylhexacene (several month).^33^ The film of 1a was grown by vacuum sublimation and exhibited a structure similar to the film in Fig. 2c (Fig. S3†). The ionisation potential (*E*_ip_) and electron affinity (*E*_ea_) of the film 1a were −5.24 and −3.30 eV, respectively, while those of 1b were −4.81 and −2.70 eV, respectively (Fig. S4†). The *E*_ip_ of thin film 1b (−4.81 eV) was consistent with that of the crystalline powder reported previously (−4.96 eV).^14^ The energy gap of 1a (1.94 eV) was less than that of 1b (2.11 eV). Theoretical computation results (DFT, B3LYP/6-31G(d) level) revealed the HOMO and LUMO of 1b to be −4.68 and −2.90 eV, respectively, while the corresponding values for 1a were −4.81 eV and −3.04 eV, respectively. The HOMO and LUMO were lowered by bromination compared with those of 1a, indicative of the electron-withdrawing effect by the bromo substituent. The HOMO–LUMO gap of 1a was 1.77 eV, whereas that of 1b was 1.78 eV, supporting the experimental results (Table 1).

**Table 1:** Physical properties of 1a–b

Sample	Ionization potential (*E*_ip_, eV)^a^	Electron affinity (*E*_ea_, eV)^b^	Energy gap (eV)^c^	Optical gap (eV)^d^	HOMO (*E*_H_, eV)^e^	LUMO (*E*_L_, eV)^e^	Energy gap (eV)^f^	Reorganization energy (*λ*^+^, meV)^e^
1a	−5.24	−3.30	1.94	1.47	−4.81	−3.04	1.77	85
1b	−4.81	−2.70	2.11	1.41	−4.68	−2.90	1.78	79

a Estimated by photoelectron yield spectroscopy.

b Estimated by low-energy inverse photoemission spectroscopy.

c *E*_ip_ − *E*_ea_.

d Estimated by the edge of absorption spectra of thin-film.

e B3LYP/6-31G(d).

f *E*_H_ − *E*_L_.

### 2.3. Charge transport properties

**Fig. 3:**
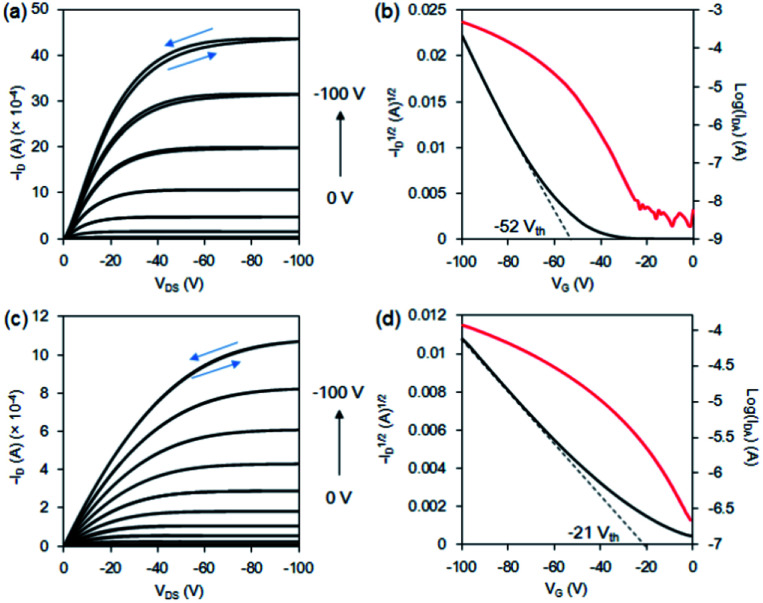
Vacuum-deposited thin-film OFET made using 1a. (a) Output characteristics, where D and S are the drain and source, respectively. (b) Transfer characteristics recorded at *V*_DS_ = −100 V, where G is the gate, and the vacuum-deposited thin-film OFET was made using 1b. (c) Output characteristics, where D and S are the drain and source, respectively. (d) Transfer characteristics recorded at *V*_DS_ = −100 V, where G is the gate.

The properties of organic field-effect transistors (OFETs) made with the films of 1a–b were examined. The OFET devices were fabricated by vacuum sublimation of 1a–b under a pressure of 8 × 10^−6^ torr to deposit the thin films on an HMDS/SiO_2_/Si substrate, followed by the deposition of gold electrodes on the top of the films. The film thickness of 1a–b was 60 nm. The channel dimension of the source/drain electrodes was 45 × 2000 μm. The output parameters were measured on a selected film across a source–drain channel, followed by the plot of drain current (*I*_D_) *versus* drain source voltage (*V*_DS_) at various gate voltages (*V*_G_). The corresponding transfer characteristics were plotted for log(*I*_D_) *versus V*_G_ at a *V*_DS_ of −100 V and *I*_D_*versus V*_DS_ in the saturation mode. The field-effect hole mobility of 1a was measured, and the mobility values ranged from 0.21 to 0.83 cm^2^ V^−1^ s^−1^ with threshold voltages of −50 to −69.3 V. The averaged performance of six independent devices was 0.52 cm^2^ V^−1^ s^−1^ and a threshold of −56.3 V. The best mobility of bromo-hexacene 1a was observed at 0.83 cm^2^ V^−1^ s^−1^ with an on/off ratio of 5.0 × 10^4^ and a threshold of −52 V (Fig. 3a–b). To compare the hole mobility, parent 1b was also tested. The mobility of 1b in the saturation mode ranged from 0.072 to 0.076 cm^2^ V^−1^ s^−1^ with a threshold voltage ranging from −19 to −22 V. The averaged performance of six independent devices was 0.074 cm^2^ V^−1^ s^−1^ and a threshold of −20.7 V. The best film mobility of 1b was observed at 0.076 cm^2^ V^−1^ s^−1^ with an on/off ratio of 2.4 × 10^2^ and a threshold of −21 V (Fig. 3c–d). Previously, the hole mobility of hexacene 1b has been reported in the single-crystal phase and in the spin-coated thin-film phase by the precursor method. The best hole-transfer mobility by spin-coated 1b was 0.035 cm^2^ V^−1^ s^−1^, with a similar surface treatment on SiO_2_/Si substrate. Although our fabrication conditions were not fully optimised, the mobility of the vacuum-deposited film was greater than that of the spin-coated one. It indicates that better crystalline films of 1b were formed by thermal deposition. The mobility of film 1a exhibited a larger range of randomness compared to 1b. However, a higher hole-transfer efficiency of 1a ranging 7- to 10-folds compared with that of 1b was observed in all tested devices. This result indicated that the packing moiety and/or film morphology possessing a better charge-transfer pathway may account for the mobility. Previously, a single-crystal bromopentacene was found to exhibit a 4-fold faster hole-transfer speed than that of non-substituted pentacene, while their reorganization energies were estimated to be 102 meV and 95 meV (B3LYP/6-31Gd level), respectively.^23^ DFT computations revealed that the reorganisation energy between the radical cation and ground state of 1a was 85 meV (B3LYP/6-31Gd level). This value was quite close to that of 1b (79 meV), suggesting a similar energy loss during structure reorganization in the hexacenes 1a and 1b.

### 2.4. TEM, XRD, and AFM measurements of hexacene films

**Fig. 4:**
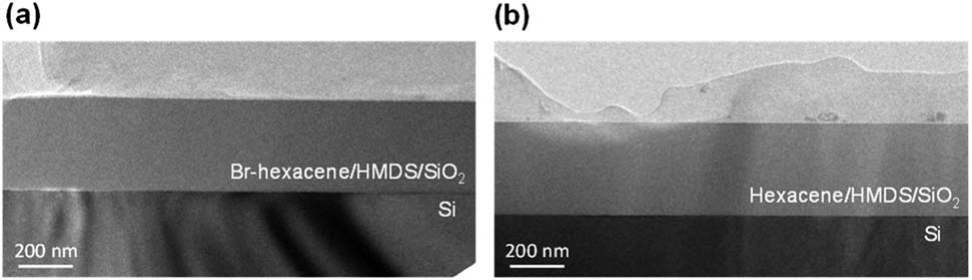
TEM image of thin films 1a (a) and 1b (b). Top: structure of films on HMDS/SiO_2_/Si.

To investigate the morphology of thin-film, we performed a surface analysis on the films 1a and 1b. Fig. 4 showed cross-section of transmission electron microscope (TEM) image of *ca.* 60 nm deposited films 1a and 1b on HMDS/SiO_2_/Si surface. The ion milling method allows us to examine a cross-section of substrate at the interface of 1a–b and HMDS/SiO_2_/Si. The TEM image show a good continuous growth of 1a and 1b films on HMDS/SiO_2_/Si surface, suggesting both films were deposited uniformly on the substrate.

The parent structure of hexacene 1b exhibited a herringbone arrangement, where the face-to-edge stacking arrangement between adjacent molecules avoided the progress of dimerization and led to a high thermal stability in the solid state.^14^ The vacuum-deposited film of 1b exhibited out-of-plane X-ray diffraction (XRD) peaks along (00*l*) direction and in-plane XRD along (*hk*0) direction (Fig. S4†).

These patterns indicated that the molecules in the deposited film are oriented vertical to the surface along their long axis. In addition, this film orientation was consistent with the reported out-of-plane pattern of heat-converted film 1b from corresponding precursor compound.^21^ The (001) peak was observed at 4.84° corresponding to an interplanar distance of 18.3 Å. This value can be compared with the *d*-spacing in the single crystal of 1b, which has been estimated to be 16.4 Å. It indicates that the molecules in the crystalline film 1b are tilted on the surface of HMDS/SiO_2_/Si substrate. The out-of-plane and in-plane XRD peaks of vacuum-deposited film 1a exhibited (00*l*) and (*hk*0) patterns, indicating that molecules in film 1a is oriented along the long axis normal to the surface (Fig. S4†). Although film 1a exhibited weaker XRD diffraction peaks compared with that of 1b, the 2*θ* angle of 1a observed at a smaller angle of 4.43° on the HMDS/SiO_2_/Si substrate. The *d*-spacing of bromohexacene molecules is estimated to be 20.0 Å. The larger *d*-spacing revealed that the molecules interact through the *a*–*b* axis.

**Fig. 5:**
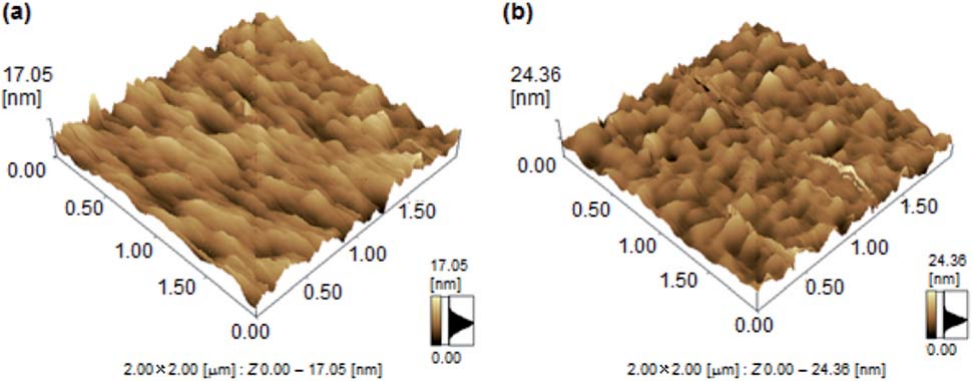
AFM images of thin films of 1a and 1b.

To further study of finding the difference of mobility between 1a and 1b, the atomic force microscope (AFM) analysis was investigated. The films 1b revealed a high surface roughness of 12.01 nm (Fig. 5). In contrast, film 1a revealed a lower roughness of 6.48 nm. This smoother surface in 1a achieved a small energy loss during the transport of holes between the source and drain.

## 3. Materials and methods

### 3.1. General information

The ^1^H and ^13^C NMR spectra were recorded in solutions on a BrukerAV600 (600 MHz) spectrometer. The ^1^H and the ^13^C NMR chemical shifts were reported as *δ* values (ppm) relative to external Me_4_Si. The coupling constants (*J*) were given in hertz. High resolution FAB mass spectra were recorded on a JMS-700 MStation spectrometer. FAB MS spectra were measured with 3-nitrobenzyl alcohol (NBA) as the matrix. Analytical thin layer chromatography (TLC) was performed on silica gel 60 F_254_ Merck. Column chromatography was performed on KANTOSi60N (neutral). Absorption and reflectance spectra were recorded on a SHIMADZU UV-3600. IR spectra were performed by SHIMADZU IRPrestige-21 spectrophotometer. AFM measurements were tested by SHIMADZU SPM-9700. The elemental analyses were recorded on a Yanaco CHN recorder MT-6. THF was distilled from sodium benzophenon ketyl. Toluene was distilled from CaH_2_. Other solvents and reagents were of reagent quality, purchased commercially, and used without further purification.

### 3.2. ^13^C CP/MAS NMR

The ^13^C CP/MAS NMR spectra were acquired with a Bruker Avance 400 MHz NMR spectrometer, equipped with a 4 mm double resonance probe operating at the ^1^H and the ^13^C Larmor frequencies of 400.13 and 100.63 MHz, respectively. For the polarization transfer, the contact-time was set to 1.75 ms. During data acquisition, ^1^H decoupling by spinal-64 was applied. Powdered samples were packed in 4 mm zirconium oxide MAS rotors with Kel-F cap. A sample spinning frequency of 12 kHz was used and regulated by a spinning controller within ±1 Hz. All CP-MAS experiments were carried out at ambient temperature. The ^13^C NMR chemical shifts are referenced to the methyl signal (=17.36 ppm) of hexamethylbenzene, which was used as an external standard. All measurements were performed in air, and the sample tube was kept in the dark between measurements.

### 3.3. FET measurement

The electrical measurements were carried out in vacuum using a semiconductor parameter analyzer (B1500A, Keysight). The saturation mobility (*μ*_sat_) was extracted from the slope of the square root of the drain current plot *vs. V*_G_ from eqn (1).1
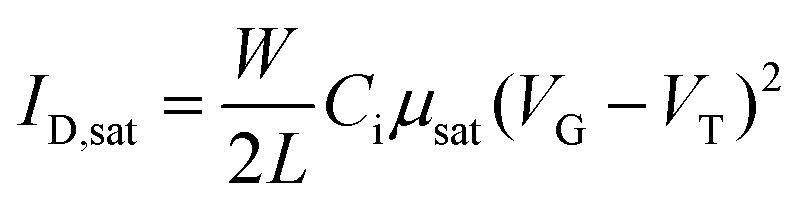
where *I*_D,sat_ is the drain-to-source saturated current; *W*/*L* is the channel width to length ratio; *C*_i_ is the capacitance of the insulator per unit area, and the *V*_G_ and *V*_T_ are gate voltage and threshold voltage, respectively. A heavily doped silicon (Si) wafer was used for a back gate electrode, which was covered with a 300 nm-thick thermally grown SiO_2_ (*C*_i_ = 10.2 nF cm^−2^) as the gate insulator. Channel length (*L*) and width (*W*) were 2000 μm and 45 μm.

### 3.4. TEM measurement

The organic films were vacuum sublimation of 1a–b under a pressure of 8 × 10^−6^ torr to deposit the thin films for 350 nm on a HMDS/SiO_2_/Si substrate. Cross-sectional TEM samples for all of the films were prepared by mechanical thinning and ion milling. The transmission electron microscope used in this study was a JEOL JEM-2100F, which was operated at an accelerating voltage of 200 kV.

### 3.5. Synthesis of materials

#### 3.5.1. Synthesis of (6 *aS*,14*aR*,15*S*)-10-bromo-17-(propan-2-ylidene)-6,6*a*,14*a*,15-tetrahydro-6,15-methanohexacene-7,14-dione 8

A solution of 1*R*,4*S*-11(propan-2-ylidene)-1,4-dihydro-1,4-methanoanthracene 3 (232 mg, 1.00 mmol), benzoquinone 4 (108 mg, 1.00 mmol), and 3,6-bis(2-pyridyl)tetrazine (248 mg, 1.05 mmol) in toluene (50 mL) was heated at 95 °C for 24 h under a nitrogen atmosphere. After the reaction, the mixture was quenched with 30% H_2_SO_4_, extracted with CH_2_Cl_2_, and the organic solution was dried over MgSO_4_ and concentrated *in vacuo*. The crude product was purified by a silica gel chromatograph eluted with CH_2_Cl_2_ to afford a mixture of *endo* and *exo* geometrical isomers of 5 (163 mg) as yellow solids. The mixture was used for next step without further purification.

The mixture of 5 (163 mg, 0.519 mmol) and zinc (600 mg) and glacial acetic acid (50 mL) was sonicated for 30 min at room temperature. After reaction, the suspension was filtered and the solution was evaporated to give the crude product. Silica gel chromatography of the crude product with CH_2_Cl_2_ and treatment with iced MeOH afforded dione 6 (114 mg). The mixture was used for next step without further purification.

A mixture of diketone 6 (114 mg, 0.361 mmol) and 4-bromophthalaldehyde 7 (76.1 mg, 0.361 mmol) was dissolved in EtOH (50 mL). The EtOH solution was bubbled by nitrogen gas for 20 min to remove oxygen. To the mixture was added 10 wt% KOH/EtOH solution (2–3 drops) in the nitrogen atmosphere and stirred 72 h at room temperature under nitrogen gas. The mixture gradually became dark, and pale-yellow powder precipitated. After reaction, the precipitate was filtered and washed with EtOH and hexane to afford *endo*-8 (86.2 mg, 17% in three steps). Pale yellow powder (EtOH). *δ*_H_ (CDCl_3_, 600 MHz) 1.23 (d, *J* = 4.8, 6H), 3.17 (s, 2H), 4.47 (s, 2H), 7.45 (dd, *J* = 6.0, 3.0 Hz, 2H), 7.76 (*J* = 9.0, 7.7 Hz, 1H), 7.78 (s, 2H), 7.82 (dd, *J* = 6.0, 2.4 Hz, 2H), 7.95 (d, *J* = 8.4 Hz, 2H), 8.24 (s, 1H), 8.53 (s, 1H), 8.61 (s, 1H). *δ*_C_ (CDCl_3_, 125 MHz) 19.7, 51.3, 52.9, 117.9, 119.0, 124.1, 125.6, 127.5, 127.9, 128.6, 131.4, 131.9, 132.2, 132.8, 132.9, 133.5, 136.0, 142.3, 143.7, 197.0, 197.8.

#### 3.5.2. Synthesis of (6*S*,15*R*)-10-bromo-17-(propan-2-ylidene)-6,15-dihydro-6,15-methanohexacene 9

To a solution of dione 8 (220 mg, 0.446 mmol) in MeOH (50 mL) and THF (50 mL) in an ice bath was added NaBH_4_ (74 mg, 1.65 mmol). After 2 h, the reaction mixture was quenched with water. The aqueous solution containing precipitates was extracted with CH_2_Cl_2_. The organic layer was washed with water, and dried over anhydrous MgSO_4_. Removal solvent gave the diol 9 (212 mg) as yellow solids. This crude compound was subjected to the next step without further purification. To a mixture of diol 9 and dried pyridine (10 mL) was added dropwise POCl_3_ (0.9 mL) at 0 °C. The resulting mixture was stirred at room temperature for 72 h, then at 80 °C for another 30 min. The mixture was poured into ice water and was extracted with CH_2_Cl_2_. The organic layer was washed successfully with 3 N HCl and brine, then was dried over MgSO_4_. The crude product was purified by a silica gel chromatograph eluted with hexane/CH_2_Cl_2_ (4 : 1) to give the aromatized compound 9 (98.8 mg, 48%) as pale yellow powder. Physical data of 9: pale yellow powder (EtOH). *δ*_H_ (CDCl_3_, 600 MHz) 1.72 (s, 6H), 5.06 (s, 2H), 7.35 (dd, *J* = 6.0, 3.0, 2H), 7.43 (dd, *J* = 9.0, 7.2 Hz, 1H), 7.70 (*J* = 6.0, 3.0 Hz, 2H), 7.72 (s, 2H), 7.77–7.79 (m, 3H), 8.08 (s, 1H), 8.12 (s, 1H), 8.19 (s, 1H). *δ*_C_ (CDCl_3_, 125 MHz) 19.8, 51.4, 113.2, 118.3, 118.4, 118.9, 119.0, 119.02, 124.9, 125.5, 126.1, 127.7, 128.3, 129.6, 131.3, 131.7, 132.2, 132.4, 145.2, 145.3, 145.6, 153.8.

#### 3.5.3. Synthesis of (6*S*,15*R*)-10-bromo-6,15-dihydro-6,15-methanohexacen-17-one 2a

A mixture of olefin 9 (200 mg, 0.433 mmol) and *N*-methylmorpholine *N*-oxide (NMO) (50% in H_2_O, 1.5 mL) in a mixed solvent of acetone (50 mL) and H_2_O (1.5 mL) was stirred at room temperature until 9 was dissolved completely. To the solution was added a few drops of OsO_4_ (4% H_2_O soln). The reaction was monitored by TLC until completion, then the mixture was quenched with 15% aqueous Na_2_S_2_O_4_. The aqueous solution was extracted with EtOAc, dried over Na_2_SO_4_, and evaporated. The product was purified by a silica gel chromatograph eluted with CH_2_Cl_2_ to give diol 10 (79 mg), which was directly used in the next step. The diol 10 (79 mg) and PhI(OAc)_2_ (120 mg) in benzene (100 mL) was stirred at 60 °C for 12 h. After reaction, the mixture was cooled in an ice bath, while white precipitates formed. The solids were collected by suction filtration to give compound 2a (59 mg, 31% in two steps) as a white powder. Physical data of 2a: m.p. 181 °C. (TGA, decomp.); IR (KBr): *ν* 1786 cm^−1^ (s, C=O); ^13^C CP/MAS NMR (12 000 rpm, 100 MHz): 54.0, 118.5, 120.6, 122.5, 123.9, 126.1, 130.5, 133.2, 135.2, 192.7.

#### 3.5.4. Synthesis of 2-bromohexacene 1a

Proper amount of precursor 2a was loaded in a glass container, and the powder was heated at 210 °C for 5 min under a nitrogen atmosphere. The color of 2a changed from white to green to give 2-bromohexacene 1a in a quantitative yield. M.p. 340 °C. (TGA, sublime); ^13^C CP/MAS NMR (12 000 rpm, 100 MHz): 123.1, 126.0; HRMS: *m*/*z* 406.0387 (M+, calcd 406.03571); EA: found, %: C, 76.2; H, 3.7. For C_26_H_15_Br. Calculated, %: C, 76.1; H, 3.7.

## 4. Conclusions

A novel hexacene precursor was successfully synthesised, which can be quantitatively converted at around 200 °C to the corresponding 2-bromohexacene. It exhibited high thermal stability over 3 months in the dark. The bromine atom affected the hexacene crystal packing and decreased the HOMO–LUMO energy gap. The thin-film of 1a was fabricated by using both spin-coating and vacuum sublimation methods, and both films exhibited exciton coupling, indicative the presence of herringbone arrangement in the polycrystalline film. The film of 1a exhibited a more efficient hole-transport property compared with that of parent 1b. Hence, film 1a exhibits a higher hole mobility of 0.83 cm^2^ V^−1^ s^−1^ than that of 1b (0.074 cm^2^ V^−1^ s^−1^). Although these hole mobility were lower than that of single crystal hexacene (4.28 cm^2^ V^−1^ s^−1^),^26^ it was comparable with the reported value of solution-processed single crystal tricyclohexylsilylethynyloctafluorohexacene (0.1 cm^2^ V^−1^ s^−1^).^31^ To the best of our knowledge, this is the first study on the charge-transport property of a stable vacuum-deposited thin-film of hexacene for electronic device. Currently, other derivatives of halogenated hexacene are prepared, and their properties related to opto-electronic devices are being examined. The results will be reported in due course.

## Author's contributions

MW designed and performed the experiments and theoretical calculations; TM, CTC, MS and SSS were synthesized, measured and analysed the materials and physical properties; TM and CA designed the devices and analysed the data; JM measured and analysed the TEM; MW and TI were measured and analysed the NMR data; MW, TM and TJC were co-wrote the manuscript; all authors gave final approval for publication.

## Conflicts of interest

There are no conflicts to declare.

## Supplementary Material

RA-008-C7RA13632C-s001
