# Supplementary material for: Synthesis and physical properties of brominated hexacene and hole-transfer properties of thin-film transistors
Source: RSC Adv. 2018 Apr 10;8(24):13259–65. doi: 10.1039/c7ra13632c (PMC9079830; doi:10.1039/c7ra13632c)
Supplement: RA-008-C7RA13632C-s001 [file RA-008-C7RA13632C-s001.pdf]

Electronic Supplementary Material (ESI) for RSC Advances.
This journal is © The Royal Society of Chemistry 2018

Supplemental Information

Synthesis and Physical Properties of Brominated Hexacene and Hole Transfer Property of Thin-film Transistor

Motonori Watanabe[a]*, Takaaki Miyazaki[b], Toshinori Matsushima,[a,c,d]*, Junko Matsuda,[a] Ching-Ting Chein[e], Masahiko Shibahara[f], Chihaya Adachi,[a,b,c,d,g] Shih-Sheng Sun[e], Tahsin J Chow[e,h]*, Tatsumi Ishihara[a,g]

[a] *International Institute for Carbon Neutral Energy Research, Kyushu University, Nishi-ku, Fukuoka 819-0395, Japan*

[b] *Education Center for Global Leaders in Molecular Systems for Devices, Kyushu University, 744 Motooka Nishi-ku, Fukuoka 819-0395, Japan.*

[c] *Center for Organic Photonics and Electronics Research (OPERA), Kyushu University, Nishi-ku, Fukuoka, Japan*

[d] *Japan Science and Technology Agency (JST), ERATO, Adachi Molecular Exciton Engineering Project, 744 Motooka, Nishi, Fukuoka 819-0395, Japan*

[e] *Institute of Chemistry, Academia Sinica, No. 128, Academia Road Sec 2, Nankang, Taipei 11529, Taiwan*

[f] *Division of Natural Sciences, Faculty of Science and Technology, Oita University,Dannoharu 700, Oita, Japan*

[g] *Department of Applied Chemistry, Faculty of Engineering, Kyushu University, Nishi-ku, Fukuoka 819-0395, Japan*

[h] *Department of Chemistry, Tunghai University, Taichung 40704, Taiwan*

Contents

1	Physical property at solution state	**S2**
2	DFT results	**S3**
3	Physical property at film state	**S4**
4	NMR chart	**S5-7**

Physical property at solution state

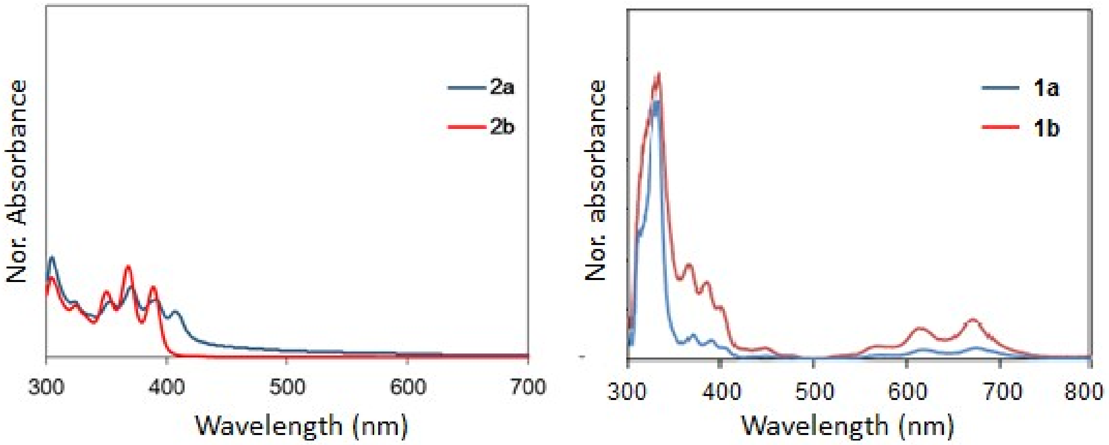

Figure S1. Absorption spectra of (left) **2a** and **2b** in 1,2,4-trichlorobenzene, and (right) after heated at 230 ^{o}C for **1a** and **1b**. Note that spectra of **1a** and **1b** should contain some decomposed species such dimerization.

DFT results

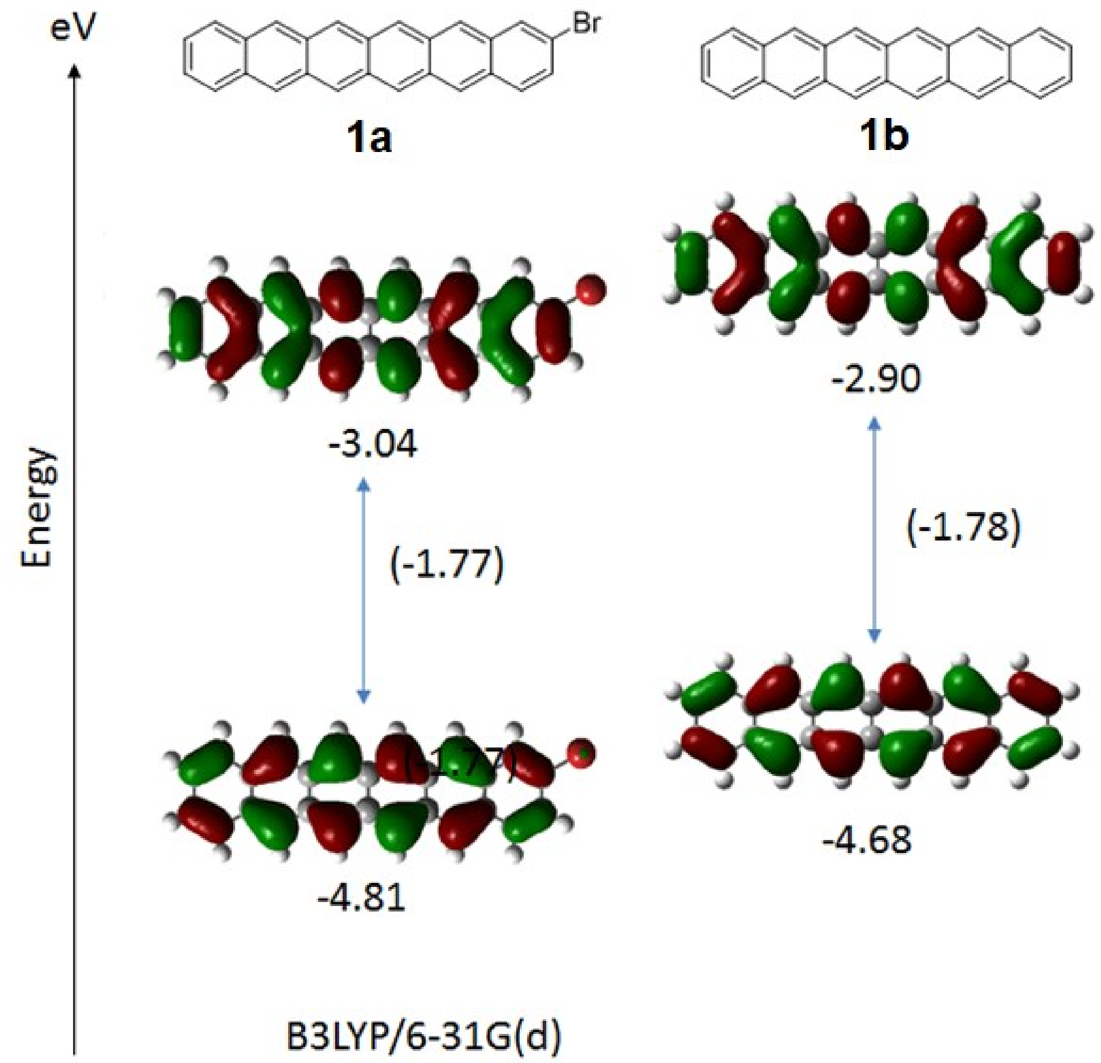

Figure S2. Energy diagram of **1a** and **1b**. The energy was estimated by B3LYP/6-31G(g) level.

Physical property at film state

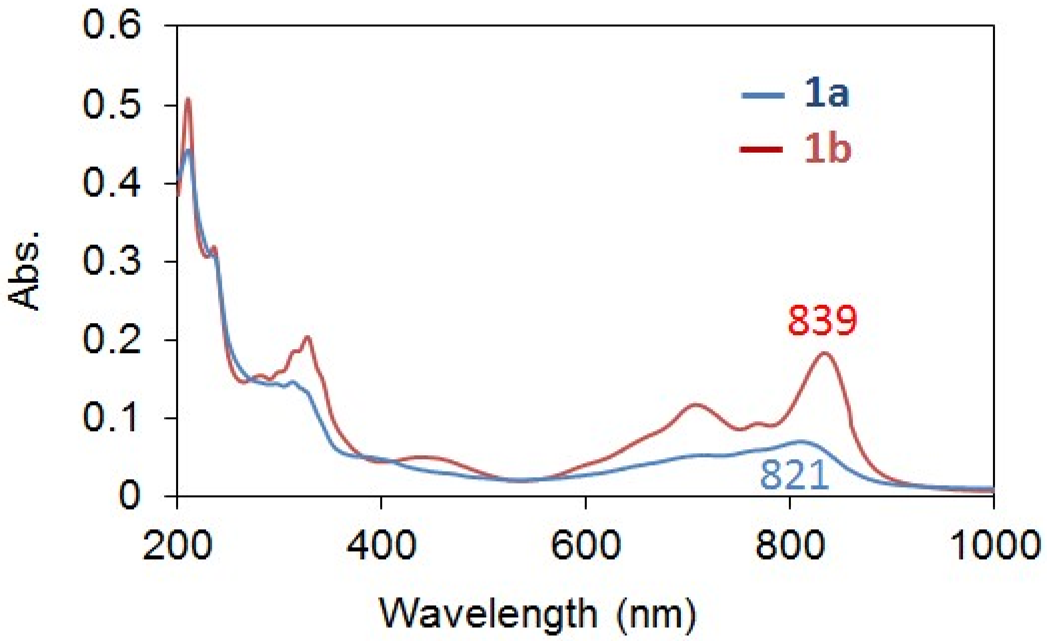

Figure S3. Absorption spectra of vacuum deposited film **1a** and **1b** on quartz plate.

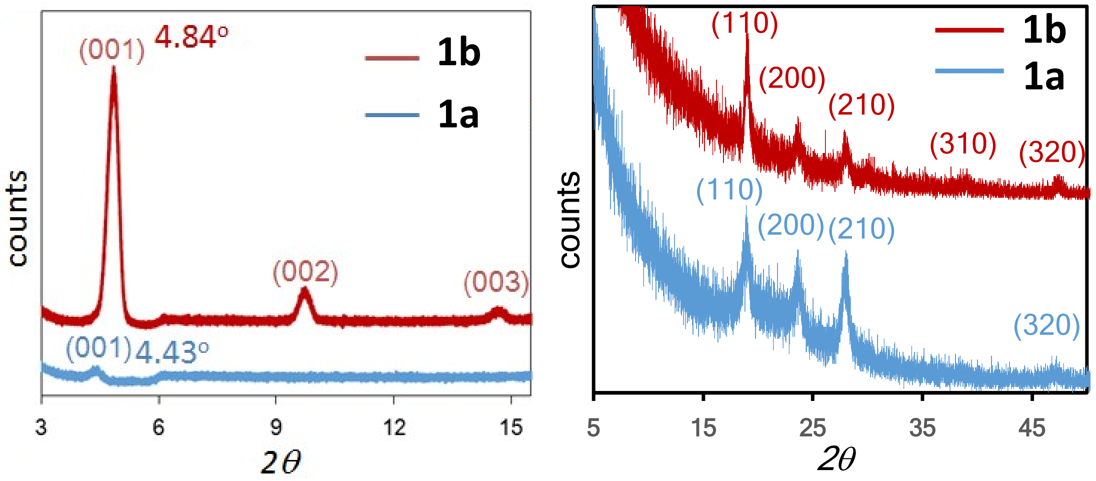

Figure S4. XRD (left: out-of-plane, right: in-plane) results of films **1a** and **1b**.

NMR spectra

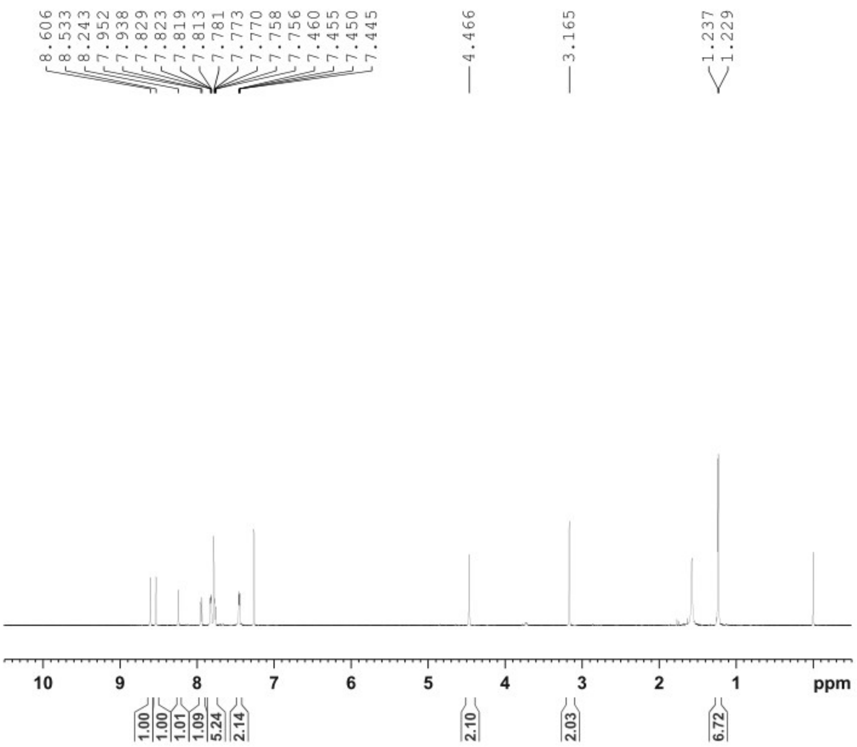

Fig S5. ^{1}HNMR spectra of **8** (600 MHz).

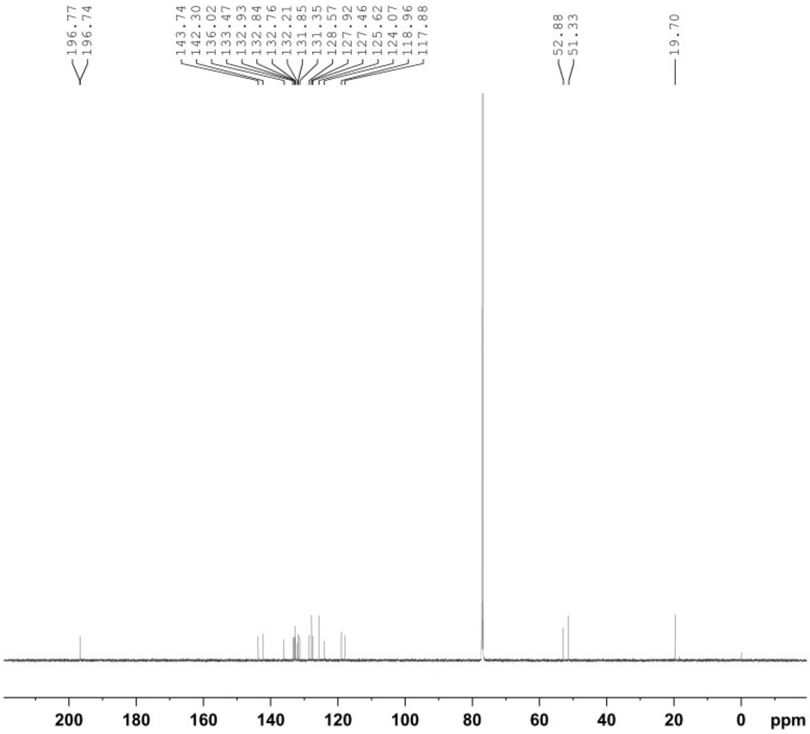

Fig S6. ^{13}CNMR spectra of **8** (125 MHz).

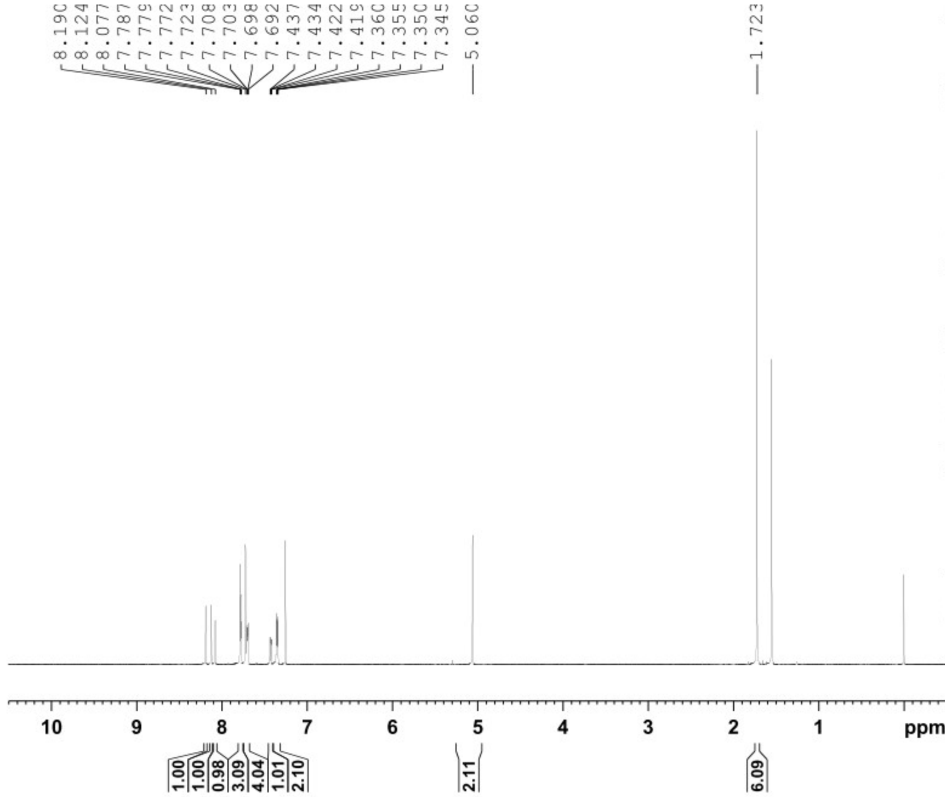

Fig S7. ^{1}HNMR spectra of **9** (600 MHz).

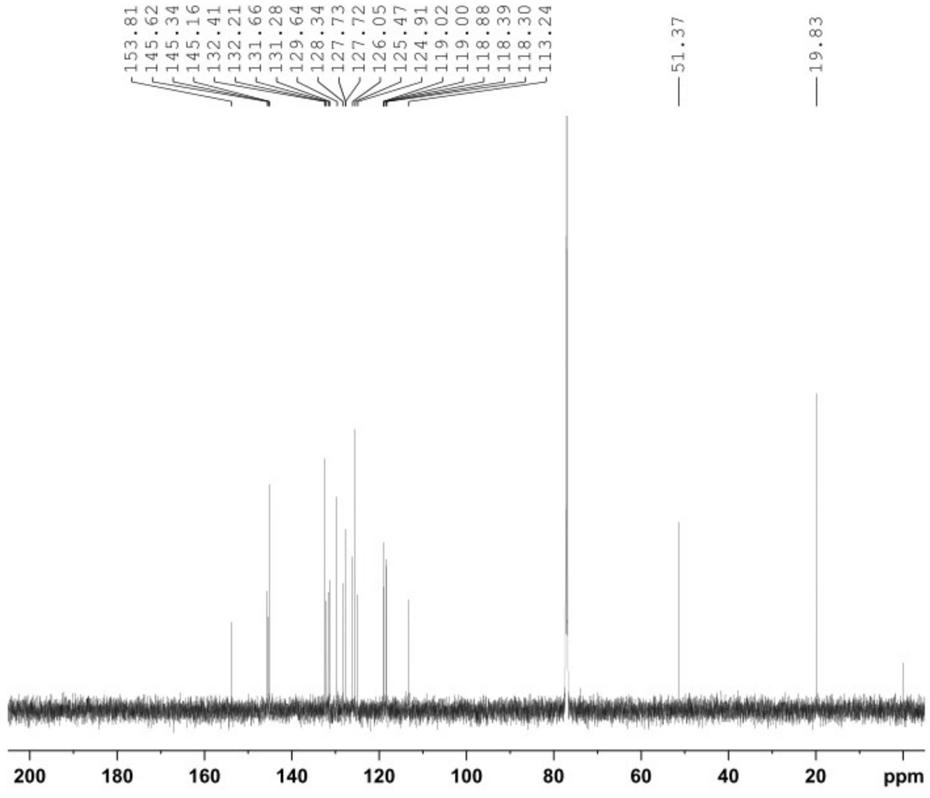

Fig S8. ^{13}CNMR spectra of **9** (125 MHz).

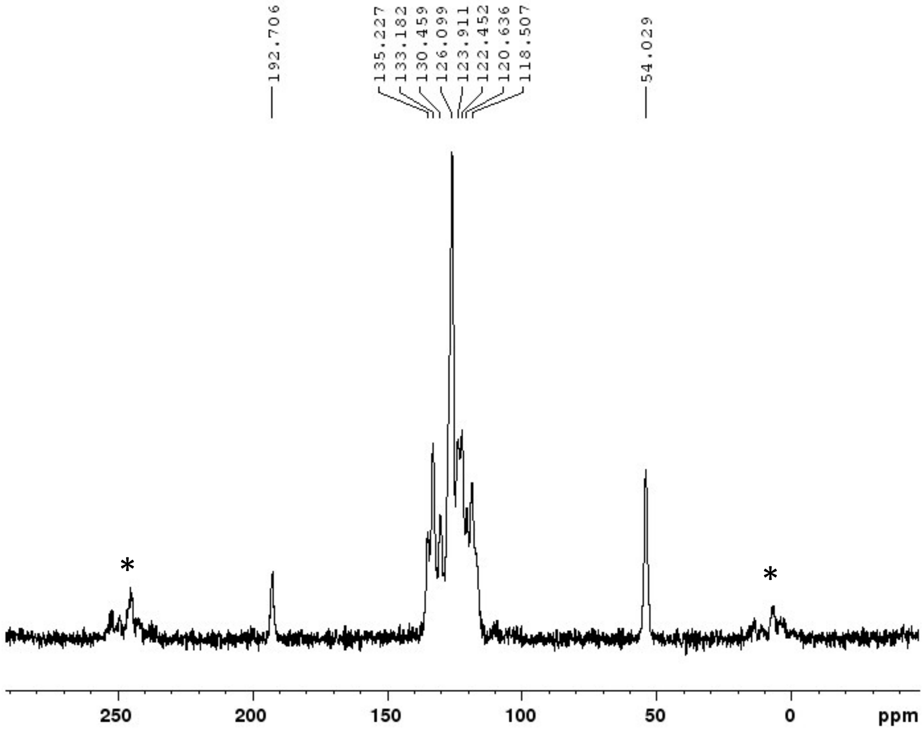

Fig S9. ^{13}C CP/MS NMR spectra of **2a** (100 MHz)

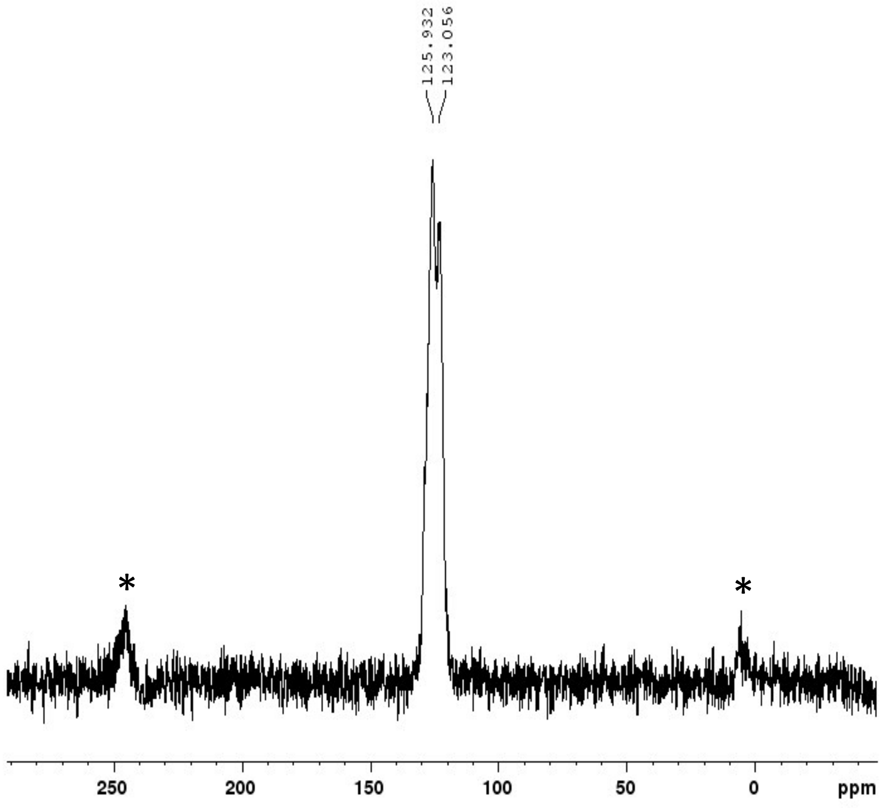

Fig S10. ^{13}C CP/MS NMR spectra of **1a** (100 MHz)
